# Evaluation of the Performance of Routine Information System Management (PRISM) framework: evidence from Uganda

**DOI:** 10.1186/1472-6963-10-188

**Published:** 2010-07-03

**Authors:** David R Hotchkiss, Anwer Aqil, Theo Lippeveld, Edward Mukooyo

**Affiliations:** 1 Tulane University, School of Public Health and Tropical Medicine, New Orleans, Louisiana USA; 2 John Snow Inc., Arlington, Virginia USA; 3 John Snow Inc., Boston, Massachusetts USA; 4 Ministry of Health, Kampala, Uganda

## Abstract

**Background:**

Sound policy, resource allocation and day-to-day management decisions in the health sector require timely information from routine health information systems (RHIS). In most low- and middle-income countries, the RHIS is viewed as being inadequate in providing quality data and continuous information that can be used to help improve health system performance. In addition, there is limited evidence on the effectiveness of RHIS strengthening interventions in improving data quality and use. The purpose of this study is to evaluate the usefulness of the newly developed Performance of Routine Information System Management (PRISM) framework, which consists of a conceptual framework and associated data collection and analysis tools to assess, design, strengthen and evaluate RHIS. The specific objectives of the study are: a) to assess the reliability and validity of the PRISM instruments and b) to assess the validity of the PRISM conceptual framework.

**Methods:**

Facility- and worker-level data were collected from 110 health care facilities in twelve districts in Uganda in 2004 and 2007 using records reviews, structured interviews and self-administered questionnaires. The analysis procedures include Cronbach's alpha to assess internal consistency of selected instruments, test-retest analysis to assess the reliability and sensitivity of the instruments, and bivariate and multivariate statistical techniques to assess validity of the PRISM instruments and conceptual framework.

**Results:**

Cronbach's alpha analysis suggests high reliability (0.7 or greater) for the indices measuring a promotion of a culture of information, RHIS tasks self-efficacy and motivation. The study results also suggest that a promotion of a culture of information influences RHIS tasks self-efficacy, RHIS tasks competence and motivation, and that self-efficacy and the presence of RHIS staff have a direct influence on the use of RHIS information, a key aspect of RHIS performance.

**Conclusions:**

The study results provide some empirical support for the reliability and validity of the PRISM instruments and the validity of the PRISM conceptual framework, suggesting that the PRISM approach can be effectively used by RHIS policy makers and practitioners to assess the RHIS and evaluate RHIS strengthening interventions. However, additional studies with larger sample sizes are needed to further investigate the value of the PRISM instruments in exploring the linkages between RHIS data quality and use, and health systems performance.

## Background

Sound policy, resource allocation and day-to-day management decisions in the health sector require timely information from routine health information systems (RHIS) in order to track the delivery of quality health care services and related support systems, including equipment and supplies, finance, infrastructure and human resources [1-5]. However, previous assessments in developing countries indicate that the RHIS is often in disarray [6]. Problems constraining RHIS performance at the country-level include: poor data quality [7,8]; limited use of available information [9,10]; weaknesses in how data are analyzed [8,11]; and poor RHIS management practices [12,13].

In addition, health system managers in developing countries tend to miss the very purpose of the RHIS - to provide data that can help track the performance of both programs and the overall health system, as the data are not typically used as part of the performance appraisal of facility staff or for the achievement of district and facility targets.

Despite the great need to improve the availability, quality and use of RHIS data at the local-level, there is a paucity of studies investigating the determinants of RHIS performance and the effectiveness of RHIS strengthening interventions. Previous studies shed light on various aspects of the RHIS but fail to provide a comprehensive picture of the RHIS, how it is organized and how various RHIS components interact with each other to influence RHIS performance. The dearth of studies in this area is likely due to a number of factors, including limited attention to RHIS as a research topic by health services researchers, the unavailability of evaluation frameworks to assess RHIS performance, inadequate research designs, and inadequate funding. Therefore, there is a clear need for placing higher priority on RHIS research and developing methodological approaches for assessing RHIS performance [6,14].

**Figure 1:**
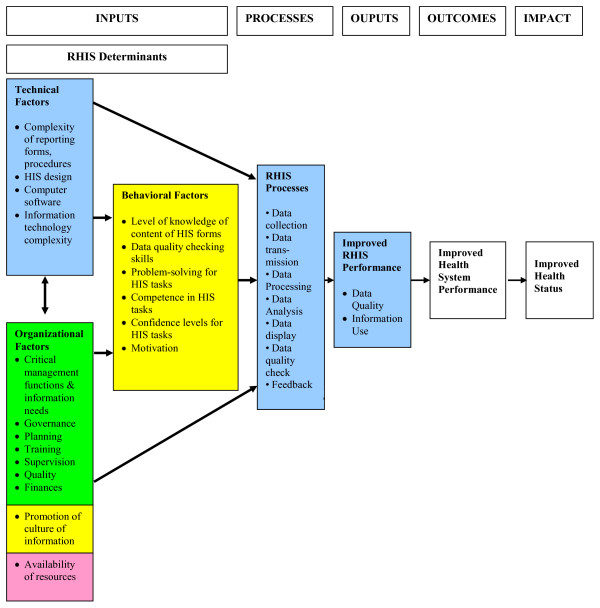
**PRISM (Performance of Routine Information System Management) conceptual framework**.

To help improve the evidence-base on RHIS performance and its determinants, Aqil, Lippeveld and Hozumi (2009) recently developed the Performance of Routine Information System Management (PRISM) framework. PRISM consists of a conceptual framework and associated data collection and analysis tools to assess, design, strengthen and evaluate RHIS [6]. As depicted in Figure 1, the conceptual framework hypothesizes that technical, behavioral and organizational determinants (inputs) influence data collection, transmission, processing, and presentation (processes), which in turn influence data quality and use (outputs), health system performance (outcomes), and ultimately, health outcomes (impact). Based on the framework, four survey instruments and associated sampling procedures and analysis guidelines were developed to assess RHIS performance, processes and technical, behavioral and organizational determinants at the facility-, district-, and country-levels.

The purpose of this paper is to assess the overall usefulness of the PRISM framework. The specific objectives are two-fold. First, we investigate the reliability and validity of the PRISM instruments, which provide measures of the determinants of RHIS performance. Second, we investigate the validity of the PRISM conceptual framework by assessing whether organizational, technical and behavioral factors are significant determinants of RHIS performance, as hypothesized.

The data for the study come from Uganda, a sub-Saharan African country that has introduced extensive health sector reforms over the past twenty years. These reforms include: further decentralization of public health care services from the central government to districts and sub-districts; increased investments in the availability and quality of primary health through a Sector-Wide Approach (SWAp); and further integration of support systems, including RHIS, as described below [15]. The process of decentralization is particularly noteworthy in Uganda, as it has meant that districts and government-run health facilities have more authority and decision-space to carryout planning and managerial roles and responsibilities, which presumably can be conducted more effectively through the use of routine health information at the local level [15,16].

In 1997, Uganda introduced the Health Management Information System (HMIS). The purpose of the HMIS is to improve on the pre-existing Health Information System (HIS), introduced in 1985, by incorporating vital management information, such as staffing levels, infrastructure, health facility management, medical equipment availability, financial information, and drug information. By integrating this information with surveillance and service delivery information already routinely collected through the HIS, the aim for the HMIS is to be "a comprehensive source of health and management information for planning, monitoring and evaluation of the health sector strategic plan. It focuses on strengthening: a) data collection and compilation of health events; b) timeliness, completeness, and accuracy of reported data: c) analysis, interpretation, and utilization for evidence-based decision-making and action; d) regular dissemination of feedback to all stakeholders; and e) enhancement of feedback to all health workers in all aspects of data management, analysis, and utilization at all levels of service delivery" [17].

The effectiveness of the HMIS in part depends on data reporting and feedback relationships as well as on trained and motivated staff at each level that properly carry out their data collection, reporting and use responsibilities. In Uganda, the government-run referral system is comprised of the following levels. The Health Centre II represents the first level of interface between the formal health sector and communities and typically provides only ambulatory services at the parish level (with a standard population of 5,000 individuals). The Health Centre III, which provides first referral cover for the sub-county (standard population of 20,000 individuals), offers basic preventive, maternal and curative care and is responsible for the support and supervision of the community and Health Centre II facilities. The Health Centre IV is a referral hospital at the county or district level (standard populations of 100,000 individuals and 500,000 individuals, respectively) which also includes on its premises the management team of the Health Sub-District Health Office. In addition to second-level referral services, the Health Centre IV provides the same types of basic services as Health Centre II and Health Centre III facilities. For tertiary services, referrals are made to regional and national hospitals. Routine data are collected in each of the types of facilities above using standardized forms issued by the Ministry of Health. This information in turn is supposed to be reported to District Health Offices and then to the Central Level Data Bank, which is operated by the Ministry of Health's Resource Centre. HMIS guidelines stipulate that feedback is to then be provided from the central level to District Health Offices, from District Health Offices to Sub-District Health Offices, and from Sub-District Health Offices to health care facilities. Uganda's HMIS collects data from both public and private sector health facilities and is probably the only example of an integrated RHIS in Africa.

According to the Ministry of Health's most recent health sector strategic plan, a number of problems limit the effectiveness of the HMIS (Republic of Uganda 2005). Data collection and reporting forms are viewed as not adequately distributed to heath care facilities and district health offices. Moreover, there is recognition that reporting forms are not properly filled and submitted, nor are data properly analyzed, fed back and utilized by the District Health Offices and health facilities for planning and managerial decision-making. The Ministry of Health also has experienced shortages of health information personnel, and the Resource Centre in Kampala has suffered from shortages of basic computers and software to facilitate the analysis of routine health data [15,17]. In the subsequent sections of this paper, we use the acronym RHIS to refer to Uganda's HMIS.

The integrated nature of Uganda's RHIS as well as the increased amount of decision-space at the district- and health facility-level make Uganda an excellent context to assess the reliability and validity of the PRISM tools as well as the validity of the PRISM framework. It is hoped that the results of the study will contribute to future RHIS assessment efforts as well as to assist Uganda's Ministry of Health to strengthen its RHIS.

## Methods

### Data

Data for the study come from health facility and staff surveys administered in Uganda in 2004 and 2007. The survey instruments used were adapted from those in the PRISM tool package [18]. The following is a summary of the instruments.

➢ **Diagnostic Tool: **This tool collects information from health care facilities and district health offices on RHIS data quality and use, RHIS procedures, supervision, information technology and user friendliness of data collection registers and reporting forms. The tool consists of a review of documents and observations of resources and displays of RHIS data.

➢ **Facility Checklist: **The facility checklist collects information from facilities and district health offices on the availability of staff, RHIS-related supplies, equipment and infrastructure.

➢ **The Management Assessment Tool: **The tool collects information through a review of documents from district health offices and health care facilities on a range of management support services, including governance, planning, training, supervision, use of performance tools, and financial resources.

➢ **The Organizational and Behavioral Assessment Tool (OBAT): **This is a self-administered tool completed by health workers at different levels on their perceptions of behavioral and organizational factors thought to influence RHIS performance. The behavioral factors include: RHIS knowledge, RHIS tasks competence, problem solving skills, confidence in carrying out RHIS tasks (self-efficacy) and motivation. The organizational factors include various questions used to assess the promotion of a culture of information within the health department.

The theoretical basis for each of the instruments is described elsewhere [6].

For the 2004 facility and district survey, which was conducted as part of a RHIS situational analysis, all six regions of the country were identified and two districts from each region (n = 12) were selected (Arua, Bugiri, Bundibugyo, Gulu, Keyniojo, Kamuli, Kumi, Luwero, Masindi, Mbarara, Mubende, and Rukungiri). The decision of which districts to be included in the study was made by officials at the Ministry of Health. Therefore, the selection of districts is purposive and not random. However, facilities in each selected district were randomly selected using Lot Quality Assurance Sampling (LQAS) methods. The sampling plan called for ten health facilities in each district to be visited: two health center II, four health center III and four health center IV facilities. Despite transportation problems and security concerns, 110 facilities were successfully interviewed (27 health center II, 48 health center III and 26 health center IV facilities), yielding a response rate of 92 percent. In 2007, the sampling plan called for revisiting all health care facilities surveyed in 2004. Of the 110 facilities surveyed in 2004,100 facilities were successfully re-interviewed, and ten facilities in which staff were unavailable were replaced by near-by facilities. Power calculations suggest that the sample size is adequate to conduct multiple regressions analyses with 25 or less independent variables [19,20].

For the self-administered organizational and behavioral assessment tool, the questionnaire was administered to one health worker (the facility in-charge) per facility in 2004 (n = 110). In 2007, the questionnaire was administered to as many as two health workers per facility in an effort to collect data from a wider variety of staff (n = 197).

It should be noted that the PRISM tool package evolved substantially between the 2004 and 2007 surveys. New questions were added to improve the measurement of several components of the PRISM framework, including RHIS data quality and use, RHIS processes, and their technical, behavioral and organizational determinants, as described below. However, all questions asked in 2004 were repeated in 2007 in order to assess changes between the two surveys, to study the utility of new questions, and to ensure that the survey instruments fit the Uganda context.

### Measures

A key measurement issue of the study concerns the multidimensional nature of most of the RHIS determinants depicted in the conceptual framework. As we describe below, most inputs of RHIS performance (technical, organizational, and behavioral factors) are measured through a series of continuous or Likert scale indicators, which are then used to generate indices following the PRISM analysis guidelines [18].

The self-efficacy scale (behavioral) incorporates four dimensions: collection, analysis, interpretation and use of data. Each dimension is based on two to four indicators, as specified in the results section. The respondents were asked to rate their self-efficacy for various RHIS tasks on a scale of zero to one hundred. For each dimension, all indicators and their ratings were added together and then divided by the total number of indicators and multiplied by one hundred to obtain a percentile score.

The scale of the index of motivation (behavioral) is based on eight items and a percentile score was calculated using the same procedure described above for the culture of information score. The scale incorporates indicators on a variety of dimensions, including perceptions of whether RHIS data are: satisfying; needed to monitor facility performance; and appreciated by fellow workers and superiors.

RHIS task competence (behavioral) was measured by a pencil and paper test that measures the ability of respondents to perform calculations, and to interpret and use RHIS results.

The promotion of a culture of information (organizational) is operationally defined as an organization having the capacity and control to promote values and beliefs among its members to promote collection, analysis and use of information to accomplish its goals and mission. For assessing whether health facilities promote a culture of information, the construct is operationalized as having five dimensions - the promotion of: 1) data quality; 2) evidence based decision making and accountability; 3) reward mechanisms for good work; 4) the use of information; and 5) efforts and activities to change things for the better. Each dimension is measured by two to eight items describing behaviors that are thought to directly or indirectly promote a culture of information. Each action statement or item related to these dimensions is assessed using a Likert scale of agreement, ranging from one (very weak) to seven (very strong). All items belonging to a specific dimension and their ratings are added together and divided by the total number items and multiplied by one hundred to create an overall percentile score.

To measure the two components of RHIS performance - data quality and the use of information - indices were constructed based on indicators common to the 2004 and 2007 surveys, and on an expanded list of indicators available in the 2007 survey only. Observation of records for checking data quality is considered to be the gold standard for measuring RHIS performance and their validity is well established [3]. To measure the availability and accuracy of RHIS data in our study, we compare the data contained in monthly RHIS reports with those of facility registrars for three types of services: the treatment of pneumonia, antenatal care, and HIV/AIDS services. For each service, percentile scores are generated to measure data availability and accuracy.

Similarly, the use of information is observed through a review of documents that verifies whether and how RHIS data were used in decision-making processes. The use of RHIS information is operationalized by a series of dichotomous indicators, including: whether RHIS information was discussed in staff meetings; whether RHIS information was used to help make decisions; whether RHIS information was used to help take follow-up actions or to refer issues for action; and whether updated information on various topics was displayed. Following the PRISM analysis guidelines, these indicators were aggregated to generate a composite continuous index of the use of RHIS information [21]. This approach gives equal weight to each of the indicators used in the index. We tested whether this assumption makes a difference in the analysis by applying principal components analysis (PCA) to generate the index. PCA is a well-established method to create summary indices using weighted sums [22]. PCA generates the weights that maximize the variance of the resulting composite index. In generating an index of RHIS data use, the advantage of the PCA approach over the simple addition approach is that it imposes fewer restrictions - the PCA approach generates weights while the simple aggregation approach is just a weighted sum where all weights are restricted to have the value of one.

For 2004, this index could not be generated because the facility diagnostic tool contained much more restricted information. Specifically, data were collected only on whether RHIS information was displayed through maps, charts and tables, and not on whether RHIS information was used in decision-making processes. To create an index of the use of RHIS information for our pooled data analysis (described below), we created a dichotomous indicator of whether a facility had on display a map, chart or table based on RHIS data at the time of the survey.

### Analysis

The internal consistency of the self-efficacy scale and the seven dimensions of the culture of information scale were estimated using Cronbach's alpha. Separate sets of Cronbach's alpha coefficients were calculated for the 2004 and 2007 samples. The test-retest reliability and sensitivity of the scale scores on self-efficacy, motivation and culture of information was assessed by conducting t-tests on the equality of the means from the 2004 and 2007 surveys. Typically, test-retest reliability is conducted by comparing the scores of each scale among a matched sample of individuals over a short time interval. However, our data were gathered three years apart and consist of individuals who may or may not be the same, but could not be matched. This prevents us from generating correlation coefficients of reliability using matched respondents. As a result, we take an alternative approach by conducting test-retest analysis based on group means, along the lines suggested by Cooke and Szumal (1993) [23]. One potential threat to the internal validity of these test-retest results is that there may have been RHIS interventions introduced during the period between the surveys that contributed to real changes in the levels of the scales investigated. We explore this issue in the discussion section.

Criterion-related validity is examined by assessing bivariate correlations among the behavioral instruments, organizational instruments, and the RHIS performance instruments described above. Correlation analyses were conducted at the individual- and facility-levels. For the facility-level analyses, the scale scores of the sample health workers were averaged for each facility to obtain facility-level scores.

In addition to bivariate analysis, multivariate analysis techniques were used to assess construct validity. Two types of models are estimated: Ordinary Least Squares (OLS) and probit models. The OLS models were estimated based on 2007 cross-sectional data, with the dependent variable consisting of the continuous index of the use of RHIS data, as described above, and the independent variables consisting of indicators of the technical, organizational, and behavioral factors described above. The probit models were based on pooled 2004-2007 data, using as the dependent variable a dichotomous variable that measures whether a table, chart or map based on RHIS data was displayed in the facility at the time of the survey. Model results were evaluated at the 1 percent, 5 percent and 10 percent levels of statistical significance. The analysis was carried out using Stata Statistical Software: Release 10 [24].

## Results

### Sample characteristics of respondents

We begin by briefly describing descriptive characteristics of sample respondents selected in both 2004 and 2007 for the self-administered organizational and behavioral questionnaire. In 2004, men were a greater percentage of the sample than women (57 percent vs. 41 percent), while the opposite was true in 2007 (48 percent vs. 52 percent), although difference in the sex composition of the two samples was not found to be statistically significant. We are unable to compare the educational level of staff across years due to differences in the response codes between the two surveys.

The mean age of the respondents was 33.8 years in 2004 and 34.0 years in 2007, indicating no meaningful difference in the age distribution between the two samples. The range of ages reported was similar in the two surveys (varying from 20 to 85 years in 2004 and from 21 to 59 years in 2007). The lack of significant differences in socio-demographic characteristics indicates that both groups were similar and that no specific characteristic need to be controlled when investigating the hypothesized relationships.

### Internal consistency

In developing the PRISM data collection instruments, face and content validity were assessed through a review and consultation with technical experts. The diagnostic tool that checks data quality and information use through record review and observation is considered to be a gold standard for assessing validity, as is the facility checklist which is used to measure the availability of infrastructure and equipment through observation. Thus, the validity of these tools is well-established. On the other hand, the reliability and validity of the organizational and behavioral assessment tool, which is comprised of scales of the promotion of a culture of information, motivation, and self-efficacy, was assessed through an analysis of internal consistency and by testing the hypothesized relationships depicted in the PRISM conceptual framework. Cronbach's alpha was used to measure the internal consistency of these scales, all hypothesized to be determinants of RHIS performance (Table 1). In exploratory research, alpha scores of 0.6 or higher are typically accepted as showing adequate reliability and alpha scores 0.7 or higher as showing high reliability [25,26].

**Table 1:** Composite indices for measuring underlying constructs of the determinants of RHIS performance, 2004 and 2007.

Composite Indicator	Questions*	Cronbach's alpha	
		
		2004	2007
**Self-efficacy scales**			
Perceived self-efficacy in analyzing data	I can calculate percentage/rate correctly I can compute data by months or year	0.83	0.87
Perceived self-efficacy in interpreting data	I can compute trend from bar chart I can compare data from bar chart	0.84	0.87
Perceived self-efficacy in using information	I can use data for identifying gaps I can use data for planning future actions I can use data for monitoring change in indicators I can use data for advocacy	0.93	0.92
Overall perceived self-efficacy	I can fill out the facility monthly report correctly I can check data accuracy I can calculate percentage/rate correctly I can plot data by months or year I can compute trend from bar chart I can use data for identifying gaps I can find discrepancy in registers and reporting forms I can compare data from bar chart I can use data for planning future actions I can use data for monitoring change in indicators I can use data for advocacy	0.95	0.95
**Culture of information scales**			
Promotion of use of RHIS information	Health department encourages staff to use data to monitor changes in health service indicators Health department encourages staff to use data monitor changes in health service indicators Health department encourages staff to use data for developing future action plans Health department encourages staff to use data for community actions	0.84	0.85
Promotion of evidence-based decision-making	Health department encourages staff/managers to check evidence before making decisions Health department makes staff accountable for their decisions and actions	0.68	0.53
Promotion of rewards for better performance	Health department encourages supervisors to reward good work Health department makes staff feel important by recognizing their work	0.70	0.63
Overall promotion of a culture of information	Health department encourages staff to check data quality Health department encourages staff/managers to check evidence before making decisions Health department inculcates value in staff that their efforts could change things for better Health department makes staff accountable for their decisions and actions Health department encourages supervisors to reward good work Health department makes staff feel important by recognizing their work Health department encourages staff to use data to monitor changes in health service indicators Health department encourages staff to use data monitor changes in health service indicators Health department encourages staff to use data for developing future action plans Health department encourages staff to use data for community actions	0.87	0.85
**Expanded culture of information scales**			
Promotion of evidence-based decision-making	Personal liking Superior's directives Evidence/facts Political interference Strategic objectives Community health needs Considering costs Considering all alternatives and their consequences RHIS data	NA	0.57
Promotion of use of RHIS information	Use RHIS data for setting targets and monitoring Feel that data collection is an important activity Rely on data for planning and monitoring set target Facilities are directed to display data for monitoring their set target Put a lot of efforts on RHIS activities	NA	0.80
Promotion of feedback	Seek feedback from concerned persons Discuss conflicts openly to resolve them Seek feedback from concerned community	NA	0.45
Promotion of problem-solving	Can gather data to find the root cause(s) of the problem Can develop appropriate criteria for selecting intervention for a given problem Can develop appropriate outcomes of a particular intervention Can evaluate whether the targets/outcomes have been achieved	NA	0.81
Promotion of a sense of responsibility	Perform duties honestly Are punctual Help each other in serving the patients/communities Feel committed in improving health status of the target population Live on their earned money (do not take bribes) Set appropriate and doable targets for their performance Are told that their efforts make a difference in improving population health status Usually document what they do Always tell the truth	NA	0.84
Promotion of accountability/empowerment	Are empowered to make decisions Are made accountable for poor performance Feel guilty for not accomplishing the set/target performance	NA	0.55
Overall perceived culture of information	All of the above	NA	0.91
**Motivation scale**	Collecting information not used for decision making discourages me Collecting information makes me feel bored Collecting information is a meaningful work for me Collecting information gives me the feeling that data is needed for monitoring facility performance Collecting information gives me the feeling that it is forced on me Collecting information is appreciated by co-workers and superiors Collecting information provides me the feeling that you have all the information to serve better your catchment area Collecting information causes me to feel that you are wasting time	0.68	0.55
**Respondents, *n***		90-100	185-192
**Facilities, *n***		110	110

*Likert scale responses consisted of seven codes (1-7) from "strongly disagree" to "strongly agree". For questions that were framed as a negative, the scale was reversed to ensure consistency with other questions included in the composite indicator.

Note: Because of missing data, the sample sizes for the indicators differ slightly.

To assess the questions on self-efficacy, the confidence level of respondents in carrying out RHIS tasks was categorized with multiple indicators under the dimensions of data analysis, data interpretation and data use. For both the 2004 and 2007 samples, the indicators for each dimension had alpha scores above 0.8, indicating a high level of reliability. Since reported self-efficacy for the tasks "data collection" and "checking data quality" are each based on a single question, alpha levels were not computed. For the overall self-efficacy scale for RHIS tasks, the alpha levels in both years are 0.95, indicating a high level of reliability.

The promotion of a culture of information is measured with a scale that includes self-reported perceptions on four dimensions: the promotion of data quality, the use of RHIS information, evidence-based decision-making and accountability, and the presence of rewards for better performance. The second block of information in Table 1 presents the results. Since the promotion of data quality was assessed with a single question, its alpha could not be calculated. With one exception, alpha scores for the remaining dimensions emerged as 0.6 or higher, indicating high reliability for both the 2004 and 2007 samples. The one exception was the alpha score for the "evidence-based decision-making" dimension based on the 2007 sample, which is 0.53. For the overall culture of information scale, the alpha levels are 0.87 in 2004 and 0.85 in 2007, indicating high reliability.

Based on the practical experience of applying the PRISM framework in Uganda [27] and Pakistan [28], additional questions for assessing the promotion of a culture of information were included in the 2007 questionnaire, allowing us to create revised indices. Changes included: omitting the dimension "rewarding better performance" due to its relative lack of specificity; and adding new dimensions on "a sense of responsibility", "accountability/empowerment", "feedback" and "problem solving". As shown in the third block of information in Table 1, the alpha levels for the scales of the overall culture of information, use of information, problem-solving and sense of responsibility dimensions are 0.8 or higher, indicating high reliability. Falling under the 0.6 threshold for adequate reliability are the alphas for the dimensions evidence-based decision-making, feedback and accountability/empowerment.

A scale was also constructed for "motivation for performing RHIS tasks". As indicated by the fourth block of information in Table 1, the alpha level for this scale is 0.68 in 2004, indicating adequate reliability. However, the comparable alpha level for the 2007 sample is 0.55, falling just below the 0.6 threshold for adequate reliability.

### Test-retest reliability and sensitivity

Table 2 presents the test-retest analysis findings for the scales of the use of information, a promotion of a culture of information, self-efficacy, motivation, and RHIS task competence. The analysis is based on indicators common to the 2004 and 2007 datasets. The use of RHIS information is measured by a dichotomous indicator of whether RHIS information was displayed in the facility at the time of the survey. The results suggest that the use of RHIS information did not change significantly from 2004 to 2007 (0.61 in 2004 and 0.51 in 2007).

**Table 2:** Test-retest comparisons of indicators of PRISM inputs and outputs, 2004 and 2007.

Variable	2004	2007	Standard Error of Difference (2004-2007)	T of Difference (p value)
**Use of information**	0.61	0.51	0.07	-1.46
	(0.49)	(0.50)		(0.15)
	110	117		
				
**Culture of information**				
Overall	61.9	75.5	1.65	8.25
	(13.60)	(13.20)		(0.00)
	100	192		
Use	64.4	78.7	1.95	7.32
	(17.10)	(15.61)		(0.00)
	107	192		
Evidence	60.3	73.1	2.00	6.40
	(17.18)	(16.02)		(0.00)
	105	192		
Reward	57.5	68.0	2.43	4.32
	(20.20)	(20.10)		(0.00)
	108	192		
				
**Self-efficacy**				
Overall	69.5	59.9	2.76	-3.48
	(20.67)	(21.86)		(0.00)
	90	185		
Analyzing	68.1	60.4	3.19	-2.43
	(24.72)	(25.90)		(0.02)
	98	185		
Interpreting	72.9	56.4	3.06	-5.39
	(21.52)	(26.08)		(0.00)
	99	185		
Using	70.8	58.5	2.82	-4.39
	(20.82)	(23.40)		(0.00)
	98	185		
				
**Motivation**	72.2	77.6	1.57	3.41
	(13.05)	(11.50)		(0.00)
	84	192		
**Tasks competency**				
Calculation	44.1	52.5	4.96	1.69
	(38.08)	(42.55)		(0.09)
	108	186		
Interpretation	21.6	73.4	8.01	6.47
	(25.79)	(81.23)		(0.00)
	109	186		
Use	2.8	21.9	3.17	12.89
	(16.51)	(30.43)		(0.00)
	108	108		

Note: Means, standard deviations (in brackets) and sample sizes are reported above for each survey. The indicator of "use of information" is measured at the facility-level. All other indicators are measured at the health worker-level. The standardized difference between the groups is defined as the difference in the means between the two surveys and scaled by the overall standard deviation.

Turning to the potential determinants of RHIS performance, the results suggest that the mean levels of the indices measuring a promotion of a culture of information, motivation to perform RHIS tasks and RHIS task competence were significantly higher in 2007 than in 2004. However, the index of perceived self-efficacy was significantly lower in 2007 than in 2004. These results show changes over time, which were picked up by the measurement tools, indicating either the measurement scales are not reliable or stable or the measurement scales are not only reliable but sensitive enough to pick up the change. We further discuss this issue in the discussion section.

**Figure 2:**
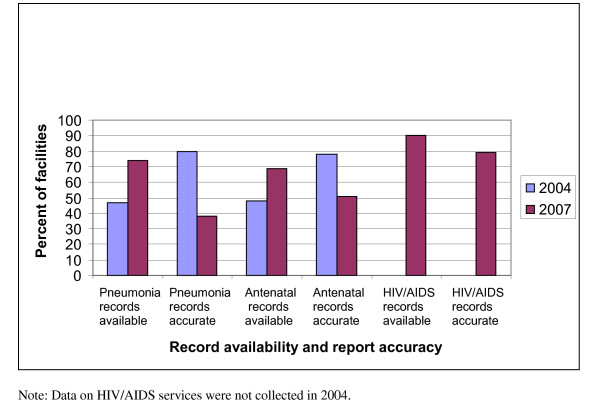
**Comparison of record availability and record accuracy by selected services, 2004 and 2007**.

Data quality and the use of information were measured through a review of existing records and reports. Was there a change in data quality? Figure 2 presents the findings of record availability, as measured by the facility having records available at the time of the survey, and data accuracy for pneumonia and antenatal care services for both 2004 and 2007. The results show that record keeping for pneumonia cases (47 vs. 74 percent) and ante-natal care cases (48 vs. 69 percent) improved substantially over time. Of those facilities where records were available, the accuracy of information reported for these selected health problems was above 75 percent in 2004. However, in 2007, when record keeping improved, accuracy was found to be substantially lower than in 2004. Before concluding that the data accuracy of the available records declined over the interval, we re-examined the data based on the assumption that all facilities with unavailable records had inaccurate data, and classified them accordingly. Based on this re-analysis, we found no statistically significant difference in data accuracy for pneumonia (χ^2 ^= 0.004741, df = 1, p = 0.95) and antenatal care records (χ^2 ^= 0.000, df = 1, p = 0.999) between 2004 and 2007.

The diagnostic tool measures the completeness of data available at the facility-level by reviewing how many data elements were filled in the monthly report of the selected month. To reduce the time needed to conduct the survey, it was decided that survey enumerators would count ten percent of the unfilled number of data elements and if the unfilled number exceeded ten percent, they would note the facility has having incomplete data. However, the instructions were not followed properly by the surveyors. Thus, it was not possible to investigate the completeness of RHIS monthly reports at the facility-level. However, district-level data on timeliness and completeness were collected in both years. A district was classified as having timely data if at least 75 percent of facilities under their authority submitted the last monthly report on time, and as having complete data if at least 80 percent of facilities under their authority submitted the monthly report for a pre-specified month (on time or not on time). The results indicate that the percent of sample districts classified as having timely data dropped (from 63 percent in 2004 to 40 percent in 2007), while the percent of districts classified as having complete data increased (from 22 percent in 2004 to 55 percent in 2007).

Using test-retest analysis, we also looked more closely at the changes in organizational determinants, behavioral determinants and RHIS performance. On average, 74 percent and 78 percent of respondents perceived that their department promotes data quality in 2004 and 2007, respectively, while on average the data accuracy levels were around 35 percent for both 2004 and 2007 (with missing records classified as inaccurate), indicating that the gap between what respondents perceived and the actual situation of data accuracy remained constant over the time interval. Similarly, the comparisons between the indicators of a promotion of a culture of information with indicators of RHIS tasks competence and observed use of information showed wide gaps in both 2004 and 2007, indicating that perceptions among the respondents that their department promotes the use of information was not aligned with actual competence to use information or observed use.

### Construct validity

To assess construct validity of the PRISM framework, we conducted bivariate analysis to investigate the hypothesized associations. Three research questions were investigated. First, do RHIS organizational factors, especially the promotion of a culture of information, affect RHIS behavioral factors? Second, does the level of confidence in performing RHIS tasks (self-efficacy) affect RHIS tasks competence? And third, are RHIS organizational and behavioral factors associated with RHIS performance, as measured by indicators of data accuracy and the use of RHIS information.

Table 3 presents Pearson correlation coefficients of the associations between indices identified through Cronbach's alpha analysis for 2007. The unit of analysis is the health worker. The results suggest that the two alternative indices of an overall culture of information are significantly associated with the RHIS tasks confidence level (self-efficacy), but not with respondents' RHIS tasks competence. Both "culture of information" indices are also found to be significantly associated with the index measuring motivation to perform RHIS tasks. In addition, there is a statistically significant association between RHIS confidence level and RHIS competence indices. These relationships are all positive, as hypothesized by the PRISM framework, and are found to be significant in both 2004 and 2007, as hypothesized in the conceptual framework, indicating construct validity. To save space, results are presented for 2007 only.

**Table 3:** Pearson correlation coefficients (p-values) of health worker-level associations between indices identified through Cronbach Alpha Analysis, 2007.

					RHIS tasks competence		
					
Index	Self-efficacy	Culture of information scale 1	Culture of information scale 2	Motivation	Ability to perform calculations	Ability to interpret results	Ability to use results
Self-efficacy	1.000	**0.214** **(0.003)**	**0.197** **(0.007)**	**0.197** **(0.007)**	**0.493** **(0.000)**	**0.361** **(0.000)**	**0.243** **(0.001)**
Culture of information scale 1		1.000	**0.708** **(0.000)**	**0.498** **(0.000)**	0.048 (0.519)	0.059 (0.420)	0.108 (0.875)
Culture of information scale 2			1.000	**0.578** **(0.000)**	0.063 (0.390)	-0.051 (0.488)	0.097 (0.193)
Motivation				1.000	**0.123** **(0.096)**	0.102 (0.166)	**0.162** **(0.030)**
Competence to perform calculations					1.000	**0.466** **(0.000)**	**0.289** **(0.000)**
Competence to interpret results						1.000	**0.364** **(0.000)**
Competence to use results							1.000

Note: Correlations that are statistically significant at the 0.10 level are indicated by **bold font**.

To assess the bivariate associations between RHIS performance and its organizational and behavioral determinants, we computed facility-level averages of the health worker-level indices found to be reliable using Cronbach's alpha analysis. We then merged these data with facility-level data on use of RHIS information and its potential determinants. The results of the analysis are presented in Table 4. Of the potential determinants of RHIS performance included in the analysis, only the self-efficacy index was found to be significantly associated with the use of RHIS information, as measured by the composite index calculated through PCA (Appendix 1). Models of the determinants of data accuracy, an indicator of data quality, were not estimated due to the very limited variation in our sample. We discuss this issue later in the paper.

**Table 4:** Pearson correlation coefficients (p-values) of facility-level associations between indices identified through Cronbach's Alpha and Principal Components Analysis, 2007.

Index	Use of RHIS information scale 2	Self-efficacy	Culture of information scale 1	Culture of information scale 2	Motivation
Use of RHIS information scale	1.000	**0.266** **(0.007)**	0.084 (0.398)	0.083 (0.402)	0.095 (0.342)
Self-efficacy scale*		1.000	**0.207** **(0.005)**	0.059 (0.548)	0.151 (0.122)
Culture of information scale 1*			1.000	**0.659** **(0.000)**	**0.434** **(0.000)**
Culture of information scale 2*				1.000	**0.556** **(0.000)**
Motivation*					1.000

*Analysis based on facility-level averages of the indices of self-efficacy, culture of information and motivation.

Note: Correlations that are statistically significant at the 0.10 level are indicated by **bold font**.

We conducted multivariate analysis to investigate the relative roles of organizational and behavioral factors on RHIS performance after controlling for other structural factors. Models were estimated using 2007 cross-sectional data and 2004-2007 pooled data. Table 5 presents the results of the OLS models of the determinants of the use of RHIS data as measured by the composite index generated through PCA, as described in Appendix 1. Models 1, 2 and 3 include as independent variables the mean self-efficacy, motivation, and culture of information indices, respectively, as well as a common set of independent variables, including: the type of health care facility; the availability of electricity; whether a RHIS assistant is on staff; the availability of a calculator; and whether a district supervisor was reported to have visited the facility in connection with RHIS activities in the quarter prior to the survey. Descriptive statistics for the variables included in the models are presented in Table S2 (Additional file 1: Table S2).

**Table 5:** Ordinary least squares model results of the determinants of the use of routine health information based on cross-sectional facility-level data, 2007.

	Model 1		Model 2		Model 3	
Independent Variable	Coefficient	SE	Coefficient	SE	Coefficient	SE
Self-efficacy index (facility-level mean)	0.017*	0.009				
Motivation index (facility-level mean)			0.007	0.020		
Culture of information index (facility-level mean)					0.004	0.016
Type of facility (= 1 if Type IV facility or hospital, = 0 otherwise)	0.312	0.418	0.629	0.397	0.623	0.397
Has electricity	-0.501	0.394	-0.545	0.395	-0.542	0.395
Has RHIS assistant on staff	1.621***	0.363	1.645***	0.365	1.649***	0.364
Has one or more working calculators	0.236	0.379	0.217	0.383	0.214	0.386
District supervisor visited facility in last quarter	0.427	0.460	0.528	0.466	0.552	0.456
Constant	-2.527***	0.710	-2.214	1.517	-2.039	1.226
Adjusted R-squared	0.212		0.183		0.183	
N	101		103		103	

*** indicates statistical significance at the 0.01 level, ** indicates statistical significance at the 0.05 level, and * indicates statistical significance at the 0.10 level

As indicated in the table, the mean self-efficacy index was found to be positive and significantly associated with the use of RHIS information at the 0.10 level of significance. The mean motivation index and the mean culture of index were also found to be positive, after controlling for other variables, but neither emerged as statistically significant. Of the other independent variables, only the presence of a RHIS assistant on the staff was found to be statistically significant.

Models of the determinants of RHIS information use were also estimated using 2004-2007 pooled data. Because the 2004 survey had fewer questions on RHIS information use compared to the 2007 survey, the dependent variable is a dichotomous indicator of whether a table, map or chart based on RHIS information was displayed in the facility at the time of the survey. The probit model results are presented in Table 6.

**Table 6:** Probit model results of the determinants of the use of routine health information based on pooled facility-level data, 2004 and 2007.

	Model 1		Model 2		Model 3	
Independent Variable	Coefficient	SE	Coefficient	SE	Coefficient	SE
Year (= 1 if 2007, = 0 if 2004)	0.691	0.452	-0.054	0.220	-0.190	0.233
Self-efficacy index (facility-level mean)	0.011	0.007				
Motivation index (facility-level mean)			-0.012	0.009		
Culture of information index (facility-level mean)					0.011	0.007
Type of facility (= 1 if Type IV facility or hospital, = 0 otherwise)	0.037	0.216	0.138	0.217	0.201	0.206
Has electricity	-0.086	0.220	-0.101	0.227	-0.149	0.215
Has RHIS assistant on staff	0.501**	0.240	0.561**	0.240	0.474**	0.243
Has one or more working calculators	0.287	0.198	0.331	0.199	0.276	0.191
District supervisor visited facility in last quarter	0.318	0.227	0.374	0.233	0.327	0.218
Constant	0.199	0.601	0.323	0.721	-1.005	0.567
Psuedo R-squared	0.066		0.068		0.058	
N	196		194		210	

Note: The dependent variable is dichotomous, measuring whether any RHIS information was displayed through maps, charts and tables at the time of the survey.

*** indicates statistical significance at the 0.01 level, ** indicates statistical significance at the 0.05 level, and * indicates statistical significance at the 0.10 level

While the index of motivation was found to be statistically insignificant at the 0.10 level, the indices of self-efficacy and a culture of information did emerge as positively associated with the use of RHIS information, as hypothesized, and are both statistically significant at the 0.11 level, just outside the 0.10 threshold level. With respect to the other independent variables, the presence of a RHIS assistant on the staff is again found to be statistically significant in each of the models. This finding is not surprising, as displaying data is presumably part of the job responsibilities of RHIS assistants.

## Discussion

The objective of this article is to investigate the reliability and validity of the PRISM framework based on sample data from health care facilities and health workers in Uganda. The framework is innovative in that it 1) stresses RHIS performance as well as organizational and behavioral determinants that typically receive inadequate treatment in the RHIS and health policy literature, and 2) includes data collection and analysis tools for empirical testing. Because previous information system frameworks do not provide tools for empirical testing [29-31], the study is the first of its kind.

Overall, the results of the study suggest that the internal consistency of the scales of the constructs for organizational and behavioral components are high, indicating that the tools are reliable for assessing RHIS tasks self-efficacy, motivation and the promotion of a culture of information. These results also suggest that these tools are sensitive and suitable for assessing changes over time, indicating that the changes between 2004 and 2007 identified through test-retest analysis are real.

In addition, the changes are internally consistent, as hypothesized by the framework. The gaps between high-perceived self-efficacy for RHIS tasks and lower observed RHIS tasks competence were expected to be filled over time, as health workers become aware of them. Therefore, it would be reasonable to expect that, over time, respondents would be become more objective in assessing their perceived self-efficacy and objective RHIS tasks competence. The results showed that was the case, as the gaps between perceived self-efficacy for RHIS tasks and objective RHIS tasks competence narrowed from 2004 to 2007. Similarly, respondents might have improved their perceptions of a promotion of a culture of information by observing that senior management had revised data collection forms and reports by including information on HIV/AIDS services and by including data collection and reporting forms that disaggregate data by age and gender to address emerging information needs of the health department. This perception along with their better RHIS tasks competence levels might have strengthened the motivation levels of respondents as well. We cannot rule out alternative explanations for these differences, such as biases that result from: survey respondents, despite having similar demographic characteristics, not being the same across the two surveys; the replacement of some facilities included in the 2004 sample with neighboring facilities in the 2007 sample; and the effect of instrumentation (getting used to tools).

Construct validity of the PRISM framework is supported by the results of the association of organizational, technical and behavioral factors with the use of RHIS information, an important dimension of RHIS performance. The promotion of a culture of information was associated with motivation, RHIS tasks self-efficacy, RHIS tasks competence, job satisfaction and use of information. Another organizational factor, the presence of dedicated RHIS staff at the facility, was found to be significantly associated with the use of information. In addition, the reliability and validity of the tools are further substantiated by the finding that data accuracy and the use of information did not change much from 2004 to 2007, which is consistent with our understanding that no major interventions were conducted in the period of time between the two surveys to ameliorate the situation.

However, the mean scores of the scales of a promotion of a culture of information, perceived self-efficacy for RHIS tasks, observed RHIS competence and perceived motivation showed significant improvement over time, indicating that the tools are sensitive to pick up changes in these factors. The training to familiarize staff with the revised forms, the addition of HIV/AIDS information to the RHIS and new provisions to disaggregate data by gender and age might have contributed to these changes. However, the size of the improvements was not large enough to affect overall RHIS performance, as measured by levels of data accuracy and the use of information. These results, of low RHIS performance and low RHIS tasks competence combined with high perceptions of promotion of a culture of information and self-efficacy for RHIS tasks, are consistent with those reported in previous assessments based on the PRISM framework in Pakistan [28,32], Mexico [33], Haiti [34], Cote d' Ivore [35] and China [36].

The study results regarding the hypothesized relationships not only support the validation of the PRISM framework but also provide insights for possible intervention strategies, as described in the conclusions section. However, that the magnitude of the size of many of the relationships investigated is small raises questions on the strength of the relationships and the potential effectiveness of interventions that operate through these mediating factors, thus potentially diluting their direct and indirect impacts.

It is to be noted that skewed responses for some of the scales of RHIS inputs and the limited variance in the indicators of RHIS performance may help explain the limited number of indicators found to be statistically significant in the analysis [37]. For example, despite finding some statistically significant associations between the use of RHIS information and selected determinants, the variation in the use of information was limited, while most of the respondents' ratings on the dimensions of a promotion of a culture of information and RHIS tasks self-efficacy were skewed positively with limited variance, which might explain why these factors were not found to be significantly significant. Moreover, the instruments that measure the promotion of a culture of information, including those on evidence-based decision-making, feedback and accountability/empowerment, need further refinement due to their low internal consistency. This might also be a possible reason for these factors not emerging as significantly significant in the models of the determinants of the use of RHIS information. In addition, we did not estimate models of the determinants of data accuracy due to the limited variation in the sample.

Based on our review of the RHIS literature, there are no RHIS studies that can be used for comparison purposes. The most relevant comparison of our results on the promotion of a culture of information, which relates to communicating beliefs and values, could be made with studies of organizational culture and communication. Clampitt and Downs (1993) showed that subordinate communication and supervisor communication has correlation coefficients of 0.17 and 0.15, respectively, with self-reported productivity [25]. Hellweg and Philips (1980) in their literature review found correlations ranging from 0.2 to 0.5 between organizational communication and productivity in organizations [38] and Pincus (1986) found similar results [39]. Thus, the study results of the promotion of a culture of information and RHIS performance are substantiated by the existing management literature. One reason for the small impact of organizational factors on performance is that these factors also act through mediating variables, and thus both direct and indirect effects are diluted. Our study results suggest that organizational factors have stronger relationships with behavioral factors such tasks competence and motivation than with overall RHIS performance, which is in line with other studies.

There are a few important limitations of the study. First, although the sample size of facilities included in the study was large enough to address the research questions (n = 110 for both the 2004 and 2007 surveys), the unavailability of RHIS records and missing information further reduced the sample size which prevented us from using more sophisticated techniques to assess the validity of the conceptual framework (i.e. fixed effects models, random effects models, factor analysis) or to conduct discriminant and convergent analyses of the subscales of a culture of information construct. Second, the 2004 dataset had only very limited information on the use of RHIS information. Only very general indicators of the display of RHIS data were available, and no indicators of the use of RHIS data in routine meetings and decision-making processes were available, which prevented us from assessing changes on these dimensions from 2004 to 2007. Because of this limitation, we believe the results of the probit model estimation, which is based on the more limited indicator of the use of RHIS information available in both 2004 and 2007, should be interpreted with caution. Third, due to problems in the administration of the survey, we were not able to assess the completeness of monthly report data at the facility-level, which along with timeliness and accuracy, is a key aspect of data quality. As a result, the relationships between data completeness and its potential determinants could not be investigated.

## Conclusions

Despite the above-mentioned limitations, the study results support the reliability and validity of the PRISM framework and its tools, indicating its utility for the policy makers, RHIS managers, professionals and RHIS designers for creating a comprehensive picture of the RHIS and identifying its strengths and weaknesses. The PRISM framework can be used for assessing RHIS performance, processes and its major organizational, technical and behavioral determinants. These tools could be applied for monitoring changes in: RHIS data quality and use of information (performance); RHIS processes and task competences; and the promotion of a culture of information. In addition, the PRISM tools could be used in research designed to evaluate the effectiveness of RHIS strengthening interventions on RHIS performance. The major interventions resulting from previous assessments based on the PRISM approach in various parts of the world include: training to improve data interpretation and use skills along with problem solving skills, which entails the use of performance improvement tools; interventions to rationalize and sometimes reduce the amount of RHIS information collected; interventions to improve the use of information technology and data warehouses; and organizational interventions aimed at establishing processes to promote the use of RHIS information through better communication of success stories and role modeling by senior management; and interventions to strengthen governance and financial resources in order to sustain RHIS activities.

Additional studies with large sample sizes are needed to investigate discriminant-convergent validity of scales measuring the promotion of a culture of information construct as well as the 'use of information' constructs. In addition, the predictive value of the PRISM framework needs to be demonstrated with further applied research in various settings. Finally, given the potentially important role that RHIS data can play in improving health systems performance, more research is needed on further improving the PRISM instruments as well as exploring the linkages between RHIS determinants, RHIS performance and health systems performance at the country- and local-levels.

## Competing interests

The authors declare that they have no competing interests.

## Authors' contributions

The study was conceived by DRH, AA and TL, designed and undertaken by DRH, AA and EM, and written by DRH and AA.

All the authors have read and approved the final manuscript.

## Appendix 1 - Index of Use of RHIS Information

The 2007 survey included a number of questions on the use of RHIS data, including whether RHIS issues and findings were discussed in staff meetings, whether facility decisions were based on RHIS data, whether there has been follow-up on these decisions, and whether various types of RHIS information were displayed in the facility through tables, charts and maps. Given that any one of these dichotomous indicators may not be sufficient to distinguish between facilities with relatively high vs. low levels of information use, summary indices were created by aggregating the indicators and through Principal Components Analysis (PCA). Because the 2004 survey include very limited information on RHIS data use, PCA analysis could not be applied to that sample. Table S1 (Additional file 1: Tables S1, S2) presents the means and standard deviations of the variables used to create the index as well as the PCA results.

The eigenvalue for the first principal component indicates the percentage of variation explained. As indicated in Table S1 (Additional file 1: Tables S1, S2), the percentage of variation explained is 45 percent for the index. The factor scores in the last column of the table, which can be interpreted as weights, indicate that each of the variables entered into the PCA is positively associated with the use of RHIS data, suggesting the variables are valid indicators of the latent variable, use of RHIS information. The PCA results were used to construct the index of the use RHIS information for the bivariate and multivariate analyses, presented in the results section.

## Pre-publication history

The pre-publication history for this paper can be accessed here:

http://www.biomedcentral.com/1472-6963/10/188/prepub

## Supplementary Material

Additional file 1 **Tables S1 and S2**. Table S1 provides the results of the Principal Components Analysis used to create an index of the use of RHIS information. Table S2 presents descriptive statistics for the variables entered in the cross-sectional model of the determinants of the use of RHIS information. Click here for file
