# Supplementary material for: Evaluation of the Performance of Routine Information System Management (PRISM) framework: evidence from Uganda
Source: BMC Health Serv Res. 2010 Jul 3;10:188. doi: 10.1186/1472-6963-10-188 (PMC2904760; doi:10.1186/1472-6963-10-188)
Supplement: Additional file 1 — Tables S1 and S2. Table S1 provides the results of the Principal Components Analysis used to create an index of the use of RHIS information. Table S2 presents descriptive statistics for the variables entered in the cross-sectional model of the determinants of the use of RHIS information. [file 1472-6963-10-188-S1.DOCX]

**Table S1: Principal components analysis results for a composite index measuring the use of RHIS information, 2007.**

| **Composite Indicator/ Indicators** | **Mean** | **Standard Deviation** | **Factor Score** |
| --- | --- | --- | --- |
| **Eigenvalue of first component=3.63, percentage of variance explained=45 percent.** | | | |
| RHIS management, such as data quality, reporting, or timeliness of reporting, discussed in a staff meeting during last three months | 0.25 | 0.43 | 0.30 |
| RHIS findings such as patient utilization, disease data, or service coverage and medicine stock-outs, discussed in a staff meeting during last three months | 0.27 | 0.45 | 0.31 |
| Decisions have been made based on last quarterly meeting | 0.25 | 0.44 | 0.37 |
| Follow-up action taken on the decisions made during the last quarterly meeting | 0.23 | 0.43 | 0.26 |
| Information displayed on maternal health (tables, charts, maps) | 0.40 | 0.49 | 0.42 |
| Information displayed on child health (tables, charts, maps) | 0.45 | 0.50 | 0.39 |
| Information displayed on service utilization (tables, charts, maps) | 0.36 | 0.48 | 0.37 |
| Information displayed on disease surveillance (tables, charts, maps) | 0.34 | 0.47 | 0.37 |

Note: Each indicator of the use of information was verified through record review and facility observation. **Table S2: Descriptive statistics for variables included in ordinary least squares model of the determinants of use of routine health information, 2007.**

|  |  | |
| --- | --- | --- |
| **Variable** | **Mean** | **SD** |
| Use of information index | 6.77e-09 | 1.905 |
| Self-efficacy index | 59.244 | 19.125 |
| Motivation index | 77.321 | 8.715 |
| Culture of information index | 75.859 | 10.995 |
| Type of facility (=1 if Type IV facility or hospital, =0 otherwise) | 0.318 | 0.468 |
| Has electricity | 0.291 | 0.456 |
| Has RHIS assistant on staff | 0.645 | 0.481 |
| Has one or more working calculators | 0.727 | 0.447 |
| District supervisor visited facility in the last three months | 0.827 | 0.380 |
| N | 110 | |
